# Reactive Energetic Plasticizers Utilizing Cu-Free Azide-Alkyne 1,3-Dipolar Cycloaddition for In-Situ Preparation of Poly(THF-*co*-GAP)-Based Polyurethane Energetic Binders

**DOI:** 10.3390/polym10050516

**Published:** 2018-05-10

**Authors:** Mingyang Ma, Younghwan Kwon

**Affiliations:** 1 Jiangxi Province Key Laboratory of Polymer Micro/Nano Manufacturing and Devices, East China University of Technology, Nanchang 330013, China; mmy861201@163.com; 2 Department of Chemical Engineering, Daegu University, Gyeongsan, Gyeongbuk 38453, Korea

**Keywords:** reactive energetic plasticizer, 1,3-dipolar cycloaddition, polyurethane, energetic binder

## Abstract

Reactive energetic plasticizers (REPs) coupled with hydroxy-telechelic poly(glycidyl azide-*co*-tetrahydrofuran) (PGT)-based energetic polyurethane (PU) binders for use in solid propellants and plastic-bonded explosives (PBXs) were investigated. The generation of *gem*-dinitro REPs along with a terminal alkyne stemmed from a series of finely designed approaches to not only satisfy common demands as conventional energetic plasticizers, but also to prevent the migration of plasticizers. The miscibility and rheological behavior of a binary mixture of PGT/REP with various REP fractions were quantitatively determined by differential scanning calorimetry (DSC) and rheometer, respectively, highlighting the promising performance of REPs in the formulation process. The kinetics on the distinct reactivity of propargyl vs. 3-butynyl species of REPs towards the azide group of the PGT prepolymer in terms of Cu-free azide-alkyne 1,3-dipolar cycloaddition (1,3-DPCA) was studied by monitoring ^1^H nuclear magnetic resonance spectroscopy and analyzing the activation energies (*E*_a_) obtained using DSC. The thermal stability of the finally cured energetic binders with the incorporation of REPs indicated that the thermal stability of the REP/PGT-based PUs was maintained independently of the REP content. The tensile strength and modulus of the PUs increased with an increase in the REP content. In addition, the energetic performance and sensitivity of REP and REP triazole species was predicted.

## 1. Introduction

Liquid polymeric binders offering a castable matrix to particulate energetic materials in irregular cases are used widely in solid propellants and plastic-bonded explosives (PBXs) [1,2]. As a processing aid, polymeric binders can give the energetic formulations better processability, and allow curing to provide improved mechanical integrity to the solid propellant and PBX composites to absorb external stimuli. Among the many polymeric binders available, hydroxy-terminated polybutadiene (HTPB) is one of the most commonly used inert binders [3,4,5,6,7]. However, considerable attention has been paid to energetic binders, because HTPB is substantially non-energetic. Glycidyl azido polymer (GAP) comprising a pendant alkyl azide group has been studied as an energetic binder because of its high density, positive heat of formation, minimal smoke, etc., [8,9,10]. As reported, GAP has relatively inferior mechanical properties at low temperatures due to the poor flexibility of the backbone [11,12,13].

Energetic plasticizers (EPs) are used as an auxiliary agent within energetic formulations to allow better processability and to improve the overall energetic performance. However, many commercially available inert plasticizers and EPs face a common challenge, i.e., how to prevent the loss of plasticizers during a lifetime under storage and working conditions. The methodology for preventing the migration and exudation of the plasticizer is of great significance to the plasticizer community. The bulk of contemporary research on preventing the migration of plasticizers is concentrated on either increasing the molecular weight and polarity of plasticizers to reduce volatility and mobility or adding nano-sized inorganic particles to bind with polar plasticizer molecules [14,15,16]. Nevertheless, the plasticizers bonding with a polymeric matrix via the intermolecular force are still mobile three-dimensionally, resulting in a loss, which makes the polymer more vulnerable. Reactive energetic plasticizers (REPs) have been recently proposed in the field of energetic formulations [17]. The ideal REPs are expected to act as common EPs during the formulation process, and as reactive plasticizers bearing chemical linking with the polymeric matrix, synchronizing the curing process to eventually prevent migration over the entire usage life. The REPs consisted of a *gem*-dinitro energetic group as the energy resource and a terminal alkyne functional group with Huisgen 1,3-dipolar cycloaddition (1,3-DPCA) reactivity. The 1,3-DPCA reaction is a powerful and appealing synthetic tool for linking two compounds [18,19,20].

In a previous report [17], GAP-based PUs were prepared in the presence of an ester linkage REPs. However, the tensile strength and modulus of REP/GAP-based PU binders had the same magnitude as the control GAP-based PU with the incorporation of REP. One way of improving the mechanical properties of GAP is to increase the flexibility of the backbone by copolymerizing with the monomers having flexible chains [11,21,22]. Thus, poly(glycidyl azide-*co*-tetrahydrofuran) (poly(GAP-*co*-THF) or PGT) with different monomer ratios was synthesized via a conventional two-step process, involving the cationic ring-opening copolymerization of epichlorohydrin and THF and its subsequent azidation with sodium azide [11,22,23]. PGT-based PUs showed a lower glass transition temperature (*T*_g_) (−65 ± 3 °C) and similar thermal stability to GAP-based PUs.

In this study, the research on REP/PGT-based PU binders was conducted with respect to plasticization, 1,3-DPCA reactivity, thermal and mechanical properties, and energetic performance, which are crucial aspects for use in energetic formulations. The miscibility and viscosity of the REP/PGT binary mixtures were evaluated at different compositions. To study the kinetics of 1,3-DPCA reaction with respect to the distinct electron-deficient alkynes towards PGT prepolymer, the reaction rate of 1,3-DPCA was assessed by tracing the ^1^H nuclear magnetic resonance (NMR) spectra and calculating the activation energy by differential scanning calorimetry (DSC), respectively. The control PGT-based PU and REP/PGT-based PUs were prepared in the presence of di-/tri-functional isocyanates to examine the effects of REPs on the thermal stability and mechanical properties.

## 2. Experimental

### 2.1. Materials

Propargyl alcohol (99%), 3-butyn-1-ol (97%), *p*-toluenesulfonic acid monohydrate (99%), epichlorohydrin (ECH, 99%), ethyl acrylate (99%), boron trifluoride tetrahydrofuran (BF_3_•THF), 1,4-butanediol (1,4-BD, 99%), sodium azide (99.5%) and 3,5-dinitro salicylic acid (98%) were purchased from Sigma-Aldrich Co. (Saint Louis, MO, USA). Chloroform (99%), ethanol (94.5%), toluene (99.8%), *N*,*N*-dimethylformamide (DMF, 99.8%), magnesium sulfate (99.5%), dichloromethane (99%), tetrahydrofuran (THF, 99.5%), sodium bicarbonate (99%), potassium hydroxide (90%) and hydrochloric acid (35.0~37.0%) were supplied by Samchun Pure Chemical Co. Ltd. (Pyeongtaek, Korea). Chloroform-d_1_ (CDCl_3_) was obtained from Merck Corp. (Darmstadt, Germany). 1,1-Dinitroethane (99%), triphenyl bismuth (98%), isophorone diisocyanate (IPDI, 98%, an isocyanate equivalent weight of 112.36 g/fn), Desmodur N-100 (98%, an isocyanate equivalent weight of 197.98 g/fn), mixture of bis(2,2-dinitropropyl)formal/bis(2,2-dinitropropyl)acetal (BDNPF/BDNPA), and mixture of bis(2,2-dinitropropyl)formal/bis(2,2-dinitrobutyl)formal (BDNPF/BDNBF) were kindly donated by the Agency for Defense Development (Daejeon, Korea).

### 2.2. Characterization

The ^1^H and ^13^C NMR spectra were recorded on a NMR spectrometer (300 MHz, Varian-Mercury, Palo Alto, CA, USA) using CDCl_3_. The chemical shifts were reported as δ ppm with tetramethylsilane (TMS) as an internal standard. The Fourier transform-infrared (FT-IR) spectra were measured using a FT-IR spectrophotometer (Nicolet 380, IetLed Co., Waltham, MA, USA). The viscosity was measured using a rheometer (MCR 301, Anton Paar Physica Co., Graz, Austria). The experiments were performed from 30 °C to 60 °C at a constant shear rate of 1.0 s^−1^ using a cone and plate (CP25-1-SN9356, dia. = 25 mm) with a gap of 0.049 mm at a heating rate of 1 °C/min. Density was measured using an Anton Paar DMA 5000 density meter (Graz, Austria). The ‘oscillating U-tube principle’ was utilized to obtain the density of the liquid. GPC spectra (Waters Corp., Milford, MA, USA) were recorded on Waters 515 HPLC pump and Waters 410 differential refractometer. Calibration curve was prepared by polystyrene standards with a series of HR-0.5 (MW range: 0~1 k), HR-1 (MW range: 100~5 k), HR-4 (MW range: 5 k~600 k) columns.

*T*_g_ was measured by DSC (DSC8000, Perkin Elmer, Waltham, MA, USA). All tests were conducted in a nitrogen atmosphere (20 mL/min) at a heating rate of 10 °C/min at temperatures ranging from −120~0 °C, and *T*_g_ of the samples was measured from the second heating scan. Thermogravimetric analysis (TGA, SDTA851^e^, Mettler Toledo, Zurich, Switzerland) was performed at a heating rate of 20 °C/min from 50 °C~400 °C at a N_2_ flow rate of 100 mL/min. The derivative thermal gravimetry (DTG) data were obtained from TGA. The decomposition temperature was the peak value obtained from the DTG curve.

The tensile strength, ultimate elongation and modulus were measured on a texture analyzer (TA-HD+1500, Stable Micro Systems Ltd., Surrey, UK) with a 50 kg_f_ load cell, TA-51 needle jig, and software (Texture Expert, Stable Micro Systems Ltd., Surrey, UK). The probe moving velocity was 0.8 mm/s. The tensile test samples were cut from the cast film with the specifications (DIN-53504, type (S2)). All tensile strengths, ultimate elongations, and initial moduli reported were the average of 3 measured values.

### 2.3. Synthesis of REPs

#### 4,4-dinitro valeric ester (DNVE)

**Scheme 1:**
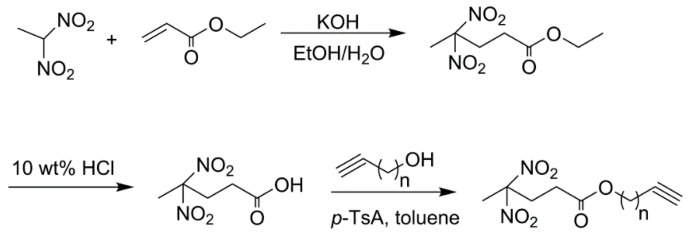
Synthetic route to REPs, PDNP (*n* = 1) and BDNP (*n* = 2).

1,1-Dinitroethane (2.4 g, 0.020 mol) and potassium hydroxide (1.2 g, 0.022 mmol) were dissolved in 100 mL of water to form the first solution. Ethyl acrylate (5.0 g, 0.05 mol) was mixed with 50 mL of ethanol and the mixture was then added slowly to the first solution. The solution was stirred at room temperature for 4 h. The reaction product was extracted by diethyl ether and distilled under reduced pressure to obtain a light-yellow liquid DNVE (see Scheme 1 for synthetic route). Yield 81.0%. ^1^H NMR (300 MHz, CDCl_3_, ppm) δ = 1.18 (s, 3H, -CH_2_-CH_3_), 2.07 (s, 3H, -C-CH_3_), 2.38 (m, 2H, -CH_2_-COO-), 2.78 (t, 2H, -C-CH_2_-CH_2_-COO-), 4.09 (d, 2H, -O-CH_2_-CH_3_). ^13^C NMR (75 MHz, CDCl_3_, ppm) δ = 170.7, 119.1, 61.5, 31.7, 28.7, 22.2, 14.1.

#### 4,4-dinitro valeric acid (DNVA)

DNVA was synthesized based on our previous work (see Scheme 1 for synthetic route) [17]. Yield: 87.1%. ^1^H NMR (300 MHz, CDCl_3_, ppm) δ = 2.15 (s, 3H, -C-CH_3_), 2.40 (m, 2H, -CH_2_-COO-), 2.76 (t, 2H, -C-CH_2_-CH_2_-COO-). ^13^C NMR (75 MHz, CDCl_3_, ppm) δ = 172.8, 120.9, 31.6, 28.7, 21.8.

#### REPs: Prop-2-yn-1-yl-4,4-dinitropentanoate (PDNP) and but-3-yn-1-yl-4,4-dinitropentanoate (BDNP)

Two types of REPs, PDNP and BDNP, were prepared according to our previous paper (see Scheme 1 for synthetic route) [17]. PDNP (*n* = 1): Yield 92.4%. ^1^H NMR (300 MHz, CDCl_3_, ppm) δ = 2.11 (s, 3H, -C-CH_3_), 2.50 (m, 2H, -CH_2_-COO-), 2.55 (m, 1H, -C≡C-H), 2.83 (t, 2H, -C-CH_2_-CH_2_-COO-), 4.67 (d, 2H, -O-CH_2_-C≡). ^13^C NMR (75 MHz, CDCl_3_, ppm) δ = 170.0, 118.8, 77.2, 75.8, 53.0, 31.6, 28.4, 22.4. BDNP (*n* = 2): Yield 91.6%. ^1^H NMR (300 MHz, CDCl_3_, ppm) δ = 2.05 (t, 1H, -C≡C-H), 2.10 (s, 3H, -C-CH_3_), 2.46 (m, 2H, -CH_2_-C≡C), 2.49 (m, 2H, -CH_2_-COO-), 2.82 (t, 2H, -C-CH_2_-CH_2_-COO), 4.16 (t, 2H, -O-CH_2_-). ^13^C NMR (75 MHz, CDCl_3_, ppm) δ = 170.5, 119.0, 80.1, 70.6, 63.1, 31.6, 28.6, 22.4, 18.9.

### 2.4. Synthesis of PGT Prepolymer

**Scheme 2:**
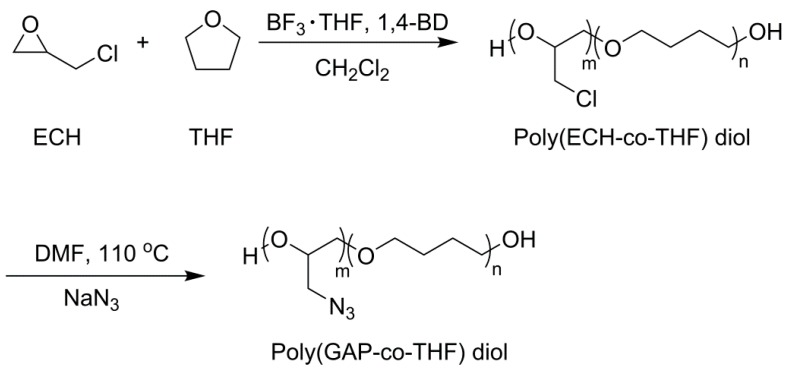
Synthetic route to poly(ECH-*co*-THF) and poly(GAP-*co*-THF) prepolymers.

BF_3_·THF (0.134 g, 0.952 mmol), 1,4-BD (0.440 g, 4.880 mmol) and dry dichloromethane (30 mL) were charged to a 100 mL two-necked round-bottom flask under a N_2_ atmosphere and mixed for 30 min at room temperature. A mixture of ECH (7.216 g, 0.078 mol) and THF (5.624 g, 0.078 mol) was then added dropwise for 6 h. After the addition, the reaction was continued for 3 h, and finally quenched by adding distilled water with stirring for 20 min. The resultant solution was neutralized with 10 wt % sodium bicarbonate solution and washed with the distilled water and dichloromethane 3 times in a 250 mL separatory funnel. The organic layer was then collected, and the solvent was evaporated under reduced pressure. The crude product was precipitated in a solvent mixture of acetone/methanol = 1/2 (*v*/*v*). The precipitated part was collected and dried in a vacuum oven at 80 °C for 24 h to give the viscous poly(ECH-*co*-THF) (see Scheme 2 for synthetic route).

Poly(ECH-*co*-THF) (8.000 g, based on 0.046 mol of ECH) in DMF (100 mL) was added into a two-neck round-bottom flask equipped with a condenser, a magnetic stirrer, and nitrogen inlet. Sodium azide (3.576 g, 0.055 mol) was added slowly and the temperature was increased to 110 °C. After 24 h of reaction time, the resultant was cooled to room temperature, filtered to remove the salts and the solvent was removed under reduced pressure. The crude product was then transferred into a 250 mL separatory funnel and extracted with the distilled water and dichloromethane 3 times. The organic layer was dried with magnesium sulfate. After complete evaporation of solvent, a viscous amber liquid was obtained (see Scheme 2 for synthetic route). ^1^H and ^13^C NMR spectra of poly(ECH-*co*-THF) and poly(GAP-*co*-THF) were shown in Figure S1 (see Supplementary Materials).

Poly(ECH-*co*-THF): Yield 80.2%. ^1^H NMR (300 MHz, CDCl_3_, ppm) δ = l.44~l.66 (m, 4H, -OCH_2_CH_2_CH_2_CH_2_O-), 3.30~3.37 (m, 4H, -OCH_2_CH_2_CH_2_CH_2_-O-CH_2_CH_2_CH_2_CH_2_O-), 3.37~3.47 (m, 2H, -OCH_2_CH_2_CH_2_CH_2_-O-CH_2_CH(CH_2_Cl)-), 3.47~3.55 (m, 2H, -OCH_2_CH_2_CH_2_CH_2_-O-CH_2_CH(CH_2_Cl)-), 3.55~3.67 (m, 8H, -O-CH_2_CH(CH_2_Cl)-O-CH_2_CH(CH_2_Cl)-), 3.85~3.95 (m, 1H, -O-CH_2_CH(CH_2_Cl)-). ^13^C NMR (75 MHz, CDCl_3_, ppm) δ = 78.4, 71.6, 71.5, 70.8, 70.7, 70.4, 70.3, 70.0, 69.7, 46.3, 44.2, 27.2, 26.9, 26.8, 26.6, 26.5, 26.4. GPC result: $\bar{M_{n}}$ = 1590 g/mol, $\bar{M_{w}}/\bar{M_{n}}$ = 1.48.

Poly(GAP-*co*-THF): Yield 87.6%. ^1^H NMR (300 MHz, CDCl_3_, ppm) δ = l.44~l.66 (m, 4H, -OCH_2_CH_2_CH_2_CH_2_O-), 3.30~3.37 (m, 4H, -OCH_2_CH_2_CH_2_CH_2_-O-CH_2_CH_2_CH_2_CH_2_O-), 3.37~3.47 (m, 2H, -OCH_2_CH_2_CH_2_CH_2_-O-CH_2_CH(CH_2_N_3_)-), 3.47~3.55 (m, 2H, -OCH_2_CH_2_CH_2_CH_2_-O-CH_2_CH(CH_2_N_3_)-), 3.55~3.67 (m, 8H, -O-CH_2_CH(CH_2_N_3_)-O-CH_2_CH(CH_2_N_3_)-), 3.67~3.80 (m, 1H, -O-CH_2_CH(CH_2_N_3_)-). ^13^C NMR (75 MHz, CDCl_3_, ppm) δ = 79.5, 72.0, 71.5, 70.7, 70.4, 70.3, 70.2, 69.8, 53.7, 52.2, 27.0, 26.8, 26.6, 26.5, 26.4. GPC result: $\bar{M_{n}}$ = 1650 g/mol, $\bar{M_{w}}/\bar{M_{n}}$ = 1.46.

### 2.5. Preparation of the PGT-Based PUs with REPs

**Scheme 3:**
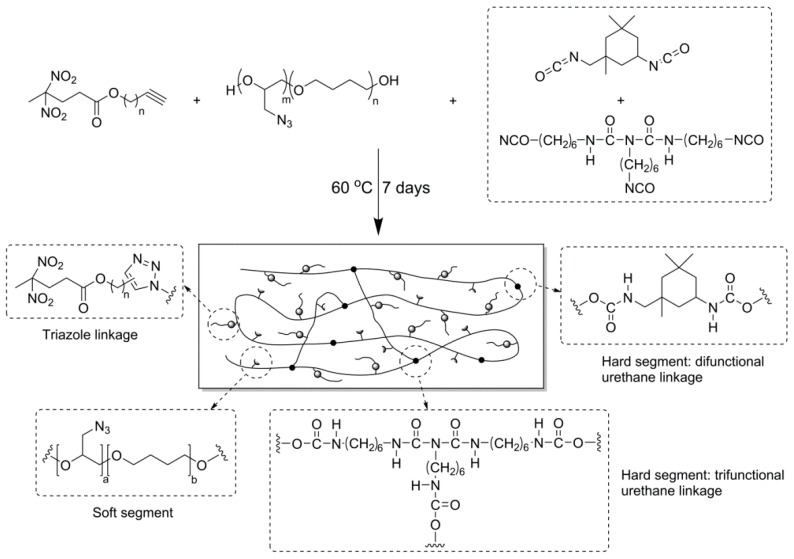
Synthetic route to the PGT-based PU in the presence of REPs.

PGT prepolymer (8.930 g) was dried at 60 °C under vacuum for 1 hr and cooled to room temperature. Subsequently, 0.632 g of trifunctional Desmodur N-100 and 0.881 g of IPDI were added rapidly to PGT. After stirring for 0.5 h at room temperature, a predetermined amount of REP based on the molar ratio of (acetylene group in REP)/(azide group in PGT), two catalysts such as triphenyl bismuth (0.03 g, 20 wt % in benzene) and 3,5-dinitro salicylic acid (0.05 g, 12.5 wt % in benzene) were added and stirred for another 0.5 h under vacuum (see Scheme 3 for synthetic route). The mixture was then cast on a Teflon coated mold (5 cm × 8 cm) and kept under vacuum at 30 °C for 3 h to remove the bubbles formed during the casting process. The curing process was carried out in an oven at 60 °C for 7 days. The control PGT-based PU was also prepared using the same procedure.

### 2.6. Cu-Free Huisgen 1,3-Dipolar Cycloaddition

The azide-alkyne 1,3-DPCA reactivity of REPs was studied with the PGT prepolymer under bulk conditions. A 1.0 g sample of the REP/PGT mixture ([-C≡CH]/[-N_3_] = 0.2/0.5) without a Cu catalyst was prepared in a vial and mixed thoroughly. The mixture was then placed in an oven at 60 °C. The sample was collected each time and dissolved in DMSO-d_6_ to measure the conversion of the 1,3-DPCA reaction using a ^1^H NMR spectrometer until the end of the reaction.

## 3. Results and Discussion

### 3.1. Plasticizing Performance of REPs

**Figure 1:**
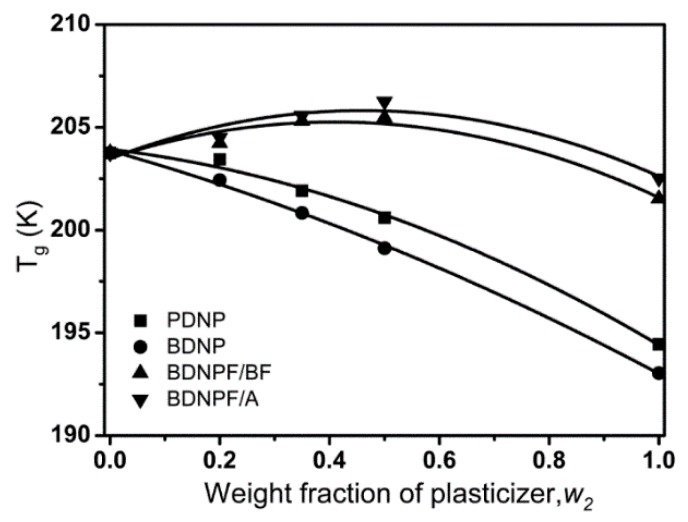
*T*_g_ variation of binary mixtures of PGT prepolymer/REPs as a function of the REP content.

As part of the criteria for evaluating the performance of the plasticizer, the miscibility and processability of the prepolymer in the presence of a plasticizer are always crucial to the fabrication of polymeric materials for the relevant applications. The *T*_g_ of the binary mixtures of PGT prepolymer and REP, containing a REP weight fraction of 0.2, 0.35 and 0.5, was measured using DSC (see Figure S2 in Supplementary Materials). As shown in Figure 1, all binary mixtures of PGT prepolymer and REP showed one *T*_g_, which was shifted inward from the *T*_g_s of the respective compounds, and the *T*_g_s of PGT prepolymer in mixtures decreased with increasing weight fraction of REPs. This result demonstrated mutual miscibility between the REPs and the PGT prepolymer. On the other hand, the extent of the shifted *T*_g_ of the binary mixtures commonly depended on the compositions and structures of the REPs. Therefore, the effect of the structures of REPs on the miscibility with the PGT prepolymer was studied quantitatively using Equation (1), which has been utilized in polymer-plasticizer binary mixtures [24].

*T*_g_ = *T*_g1_*w*_1_ + *T*_g2_*w*_2_ + *I w*_1_*w*_2_ (1)

where *w*_1_ and *w*_2_ are the weight fractions of PGT prepolymer and REP, and *T*_g1_ and *T*_g2_ are the *T*_g_ of the PGT prepolymer and REP, respectively. *T*_g_ is the measured *T*_g_ of a binary mixture at a certain composition. *I* is called the interaction parameter, and a lower *I* value means a better ability to decrease the *T*_g_ of the PGT prepolymer [24]. The *I* value was obtained by fitting the experimental data with a standard deviation, and is summarized in Table 1. The *I* value of the binary mixture was 6.31 ± 0.34 K for PDNP (*n* = 1) and 3.01 ± 0.33 K for BDNP (*n* = 2). In terms of analogous species, according to free volume theory, an increase in chain length or molecular weight was associated with an increase in free volume and a larger free volume resulted in more flexible chains [25,26]. REP with a longer spacer length (*n* = 2, larger free volume) exhibited a lower *I* value than the one with *n* = 1, implying better miscibility of the binary mixtures.

To compare the extent of miscibility between REPs and commercial EPs with uncured PGT prepolymer, the miscibility between EPs and PGT prepolymer was also quantified using the commercially available *gem*-dinitro EPs, BDNPF/A and BDNPF/BF. As shown in the Supplementary Materials (Figure S2), mixtures of EPs and PGT prepolymer were miscible for all relevant ranges, showing one *T*_g_, which was inward-shifted from each compound. Both binary mixtures of PGT:BDNPF/A and PGT:BDNPF/BF exhibited similar *I* values, at 11.04 ± 0.56 K and 10.52 ± 0.50 K, respectively, which were higher than those for the PGT:REP systems. This suggested that the REPs are more miscible with the PGT prepolymer than EPs.

The viscosity reduction of the PGT prepolymer was studied using a mixture of PGT prepolymer and REP (50:50 *w*/*w*) at elevated temperatures ranging from 30 to 60 °C (see Figure S3 in Supplementary Materials). As shown in Table 1, the addition of REPs decreased the viscosity of PGT prepolymer from 1485 to 350~450 cP at 30 °C and from 306 to 114~140 cP at 60 °C, which conformed to the temperature-dependent rheological behavior, suggesting that the REPs work properly as common plasticizers. On the other hand, the conventional EPs decreased the viscosity of the PGT prepolymer from 1485 to 648~692 cP at 30 °C and from 306 to 142~160 cP at 60 °C (see Figure S4 in the Supplementary Materials). Based on the rheological results between PGT prepolymer/REPs and PGT prepolymer/EPs systems, REPs exhibited a more effective reduction of the viscosity of PGT prepolymer. Therefore, it was conceivable that REPs could fulfil the basic requirement as a general plasticizer.

### 3.2. Huisgen 1,3-DPCA Reactivity

To chemically link the REPs with PGT-based PU binders during PU reaction, the catalyst-free Huisgen 1,3-DPCA of an organic azide and alkyne was considered to be a proper reaction, due to its ideal compatibility and specificity (byproduct-free) towards hydroxyl-telechelic azido energetic binders cured by isocyanate agents [18,19,20]. Nevertheless, the reactivity of 1,3-DPCA reaction should be controlled to manipulate the fabrication process for a propellant or PBX formulation [20]. For example, Min and coworkers studied the Cu-free 1,3-DPCA reactivity of propiolate (HC≡C-COOR) and propargyl (HC≡C-CH_2_-OCOR) species by checking the gel time when mixed with GAP. Propiolate species could form the gel within 10 min at 20 °C, and propargyl species required 9 h at 50 °C. These results displayed that the alkyne of the propiolate species was too reactive to be used in a propellant or PBX formulation. In addition, Cu(I)-catalyzed Huisgen 1,3-DPCA was excluded because of its prompt reactivity with respect to pot life-dependent situations. Cu(I) also formed a highly sensitive explosive together with the dinitramide anion when using ammonium dinitramide in actual applications [27]. Therefore, the Cu-free and medium electron-deficiency alkyne moiety were prerequisites for the present study.

**Figure 2:**
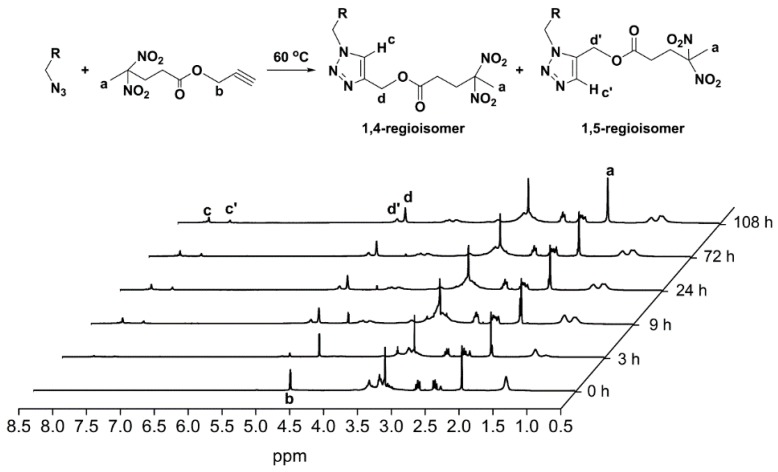
^1^H NMR spectra as a function of the reaction time of Cu-free 1,3-DPCA reaction of PDNP (*n* = 1) and PGT prepolymer carried out under the bulk condition at 60 °C. The R group in the reaction scheme above is the backbone of the PGT prepolymer.

The 1,3-DPCA reactivity between REPs and PGT prepolymer (molar ratio, [C≡C]/[N_3_]=0.2/0.5) was monitored by ^1^H NMR spectroscopy as a function of the reaction time at 60 °C, which was the same as the curing temperature. Figure 2 presents a series of representative ^1^H NMR spectra of the reaction process between PDNP (*n* = 1) and the PGT prepolymer (BDNP (*n* = 2), series was shown in Figure S5 in Supplementary Materials). The azide group was referred to as an ambiphilic dipole bearing the highest occupied molecular orbital (HOMO) and lowest unoccupied molecular orbital (LUMO) two-way interaction with dipolarophile. In this case, the HOMO of the dipole could pair with the LUMO of the dipolarophile. In addition, the HOMO of dipolarophile could pair with the LUMO of the dipole. A study on the typical Cu-free azide-alkyne Huisgen 1,3-DPCA in terms of the propargyl moiety showed that the purely thermal Huisgen cycloaddition gave rise to the 1,4- and 1,5-regioisomers [20,28]. This arose because the energy gap in either HOMO_dipole_-LUMO_dipolarophile_ or HOMO_dipolarophile_-LUMO_dipole_ was similar.

**Figure 3:**
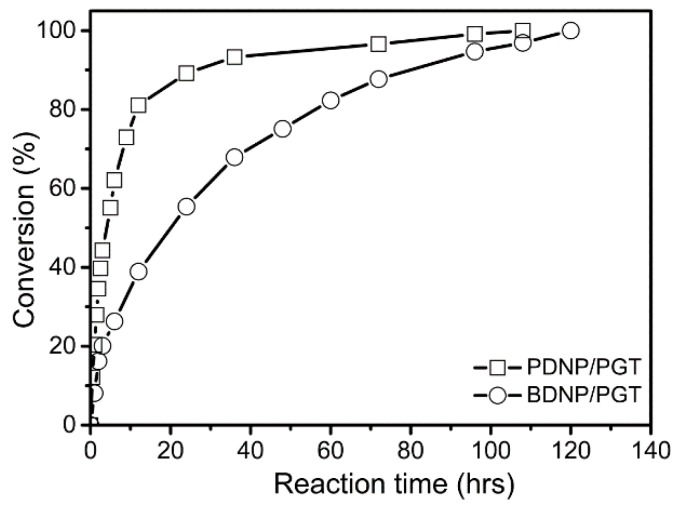
Conversion plot for Cu-free azide-alkyne 1,3-DPCA reaction of REPs/PGT prepolymer at 60 °C.

The formation of a triazole group as a result of Huisgen 1,3-DPCA reaction is identified in Figure 2, where the proton resonances of 1,4- and 1,5-regioisomers of the triazole were visible at 8.08 (c) and 7.75 ppm (c’), respectively. The proton resonances of the newly formed methylene of PDNP moieties adjacent to the triazole rings were also observed at 5.26 (d’) and 5.12 ppm (d), respectively. The methyl group of PDNP at 2.11 ppm (a) was selected as an internal standard for tracing the extent of the 1,3-DPCA reaction. The 1,3-DPCA conversion calculated by monitoring the ratio of integral areas of protons derived from methylene of PDNP at 4.67 ppm (b) is shown in Figure 3. Figure 3 shows that the Cu-free 1,3-DPCA reaction between the REPs and PGT prepolymer was complete within 5 days, which was less than the 7-day curing time of general PU binders in energetic formulations. PDNP (*n* = 1) had a higher 1,3-DPCA reaction rate than BDNP (*n* = 2), indicating that the amount of methylene between the electron-withdrawing group (EWG) and the alkyne had a great influence on the LUMO level of alkyne moiety that determined the Cu-free 1,3-DPCA reactivity [29,30]. The inductive effect caused by the methylene spacers between the EWG and alkyne groups made PDNP (*n* = 1) a more electron-deficient alkyne (a low-lying LUMO) than BDNP (*n* = 2). This could be proven from the degree of the deshielding effect on the alkynyl proton of the REPs in the ^1^H NMR chemical shift. The proton resonance of the terminal alkynyl hydrogen of BDNP (*n* = 2) appeared at 2.05 ppm, and that of PDNP (*n* = 1) was shifted downfield to 2.55 ppm, suggesting that the alkynyl proton of PDNP was supposed to be more electron-deficient.

**Figure 4:**
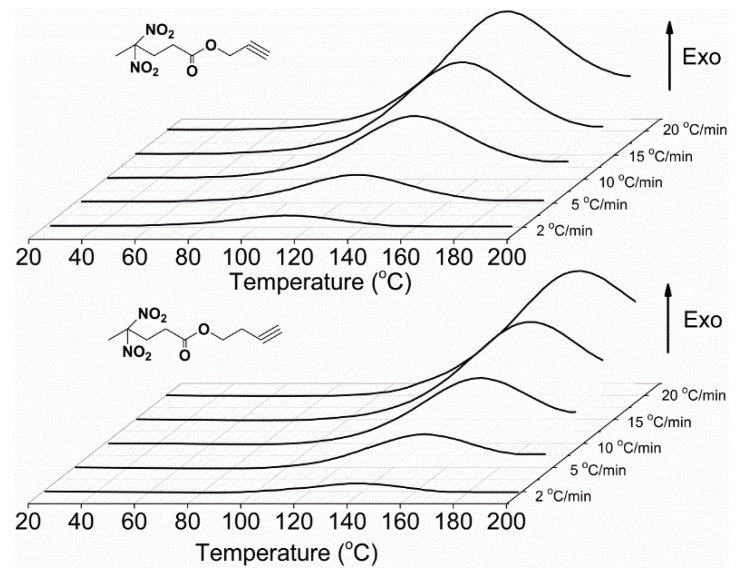
Dynamic DSC curves of PDNP (*n* = 1)/PGT (**top**) and BDNP (*n* = 2)/PGT (**bottom**) mixtures at different heating rates.

**Figure 5:**
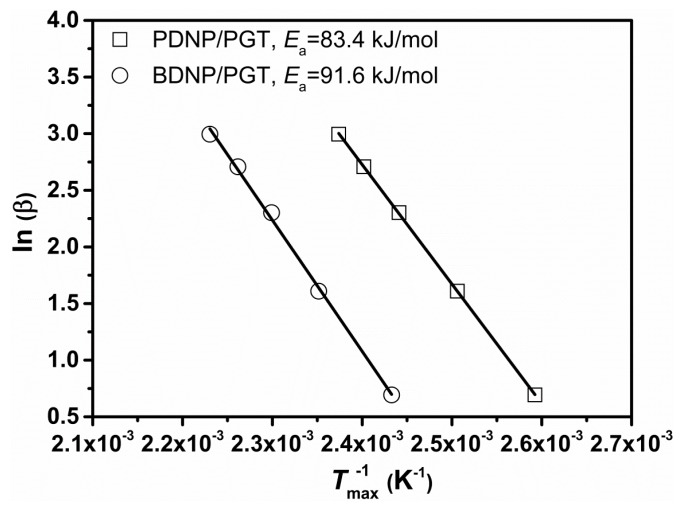
Arrhenius plots of ln(*β*) versus $T_{\max}^{-1}$ of REP/PGT and REP/GAP. For all samples, the linear fitting coefficient of determination (*R*^2^) was greater than 0.99.

The relative reactivity of REPs with the PGT prepolymer under Cu-free conditions was also investigated by estimating the *E*_a_ due to the exothermic behavior of Huisgen 1,3-DPCA. The *E*_a_ of the 1,3-DPCA reaction was obtained from Equation (2) based on ASTM E698.

(2) $$\ln(\beta )=A-\frac{1.052\;E_{a}}{R\;T_{\max}}$$

where *β* is the scan rate (K/min), *A* is a constant, *E*_a_ is the activation energy (J/mol), *T*_max_ is the temperature at the maximum exothermic temperature (K) and *R* is the universal gas constant (8.314 J/mol·K). Figure 4 shows the dynamic DSC curves of the PGT prepolymer with PDNP (*n* = 1) and BDNP (*n* = 2), respectively, at various heating rates of 2, 5, 10, 15, and 20 °C/min. In each series of measurements, the *T*_max_ shifted to a higher temperature with increasing heating rate. At the same heating rate, however, PDNP (*n* = 1) showed a lower *T*_max_ than BDNP (*n* = 2), suggesting a faster 1,3-DPCA reaction. The *E*_a_’s were calculated from the slope constructed by linear plots of ln(*β*) versus $T_{\max}^{-1}$, and found to be dependent on the number of methylene spacers of REPs. As shown in Figure 5, PDNP (*n* = 1) showed a lower *E*_a_ (83.4 kJ/mol) than BDNP (*n* = 2) (91.6 kJ/mol) indicating a relatively higher reactivity of the former. This result was in good agreement with the aforementioned 1,3-DPCA reactivity study, and the calculated *E*_a_ value in terms of propargyl species matched well with the reported ones [28,31].

### 3.3. Properties of REP/PGT-Based PUs

**Figure 6:**
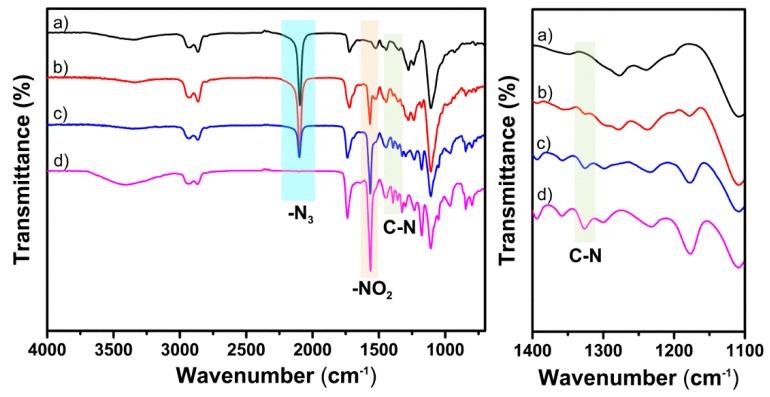
FT-IR spectra of the PDNP/PGT-based PUs in terms of [C≡C]/[N_3_] (mol/mol): (**a**) 0/0.5, (**b**) 0.1/0.5, (**c**) 0.3/0.5 and (**d**) 0.5/0.5.

The energetic binder constituents, although accounting for a relatively minor portion (10~15 wt %) in the PBX formulations, offer an appealing platform for the design and synthesis of novel energetic binders or additives, as well as for efficient control of the processability, thermal stability, mechanical properties, etc. Therefore, PGT-based PUs with the incorporation of REPs were prepared by PU reaction, along with Cu-free 1,3-DPCA reaction. The mole ratio of Desmodur N-100 and IPDI was controlled to be 2.5/7.5. The REP contents in the PGT-based PUs were controlled based on the molar ratio of (alkyne)/(azide) = 0.1/0.5, 0.3/0.5, and 0.5/0.5, respectively. Owing to the good compatibility between the 1,3-DPCA reaction and the PU reaction verified by the synchronous cure system, the curing time was of great reliability, typically within 5~7 days under mild conditions [19,32]. The resulting structures of the REP/PGT-based PUs were analyzed by FT-IR spectroscopy. Figure 6 shows the representative FT-IR spectra of the PDNP/PGT-based PUs (the BDNP series is shown in Figure S6 in Supplementary Materials). The strong absorption band at 2100 cm^−1^ was attributed to the stretching vibration of the azide group, and the stretching vibration of the nitro group appeared at 1562 cm^−1^ [9,33]. Furthermore, FT-IR spectra of the PDNP/PGT-based PUs showed a triazole ring stretching (C–N) vibration at 1330 cm^−1^ [34]. The FT-IR spectra of the PUs were devoid of a strong band at 2275 cm^−1^ belonging to NCO group, suggesting that the polymerization and 1,3-DPCA reaction proceeded simultaneously under the same reaction conditions.

The TGA data of the pristine PGT-based PU and REP/PGT-based PUs were collected at a heating rate of 20 °C/min in a nitrogen atmosphere (see Figures S7 and S8 in Supplementary Materials). Table 2 lists the thermal properties, such as the 5 wt % weight loss temperature (*T*_d,5wt__%_) and the temperature at the maximum rate of thermal decomposition of the major weight loss (*T*_d,max_). The *T*_d,5wt %_ of the REPs was observed, ranging from 170 to 176 °C with a *T*_d,max_ in the range of 230~240 °C, indicating lower thermal stability than the PGT prepolymer (*T*_d,5wt__%_ at 241 °C and *T*_d,max_ at 248 °C). On the other hand, the early weight loss from 170 to 176 °C of the REPs/PGT-based PUs was absent, regardless of the REP content, suggesting that REPs were completely attached to the side chain of PGT-based PU through the formation of a triazole ring. In addition, the early weight loss of pure REPs was just evaporation due to the low molecular weight of the REPs. Although more azide groups were converted to the more thermally stable 1,2,3-triazole moiety with increasing REP content, the prompt decrease in weight loss of all binders around 230~240 °C was still observed, particularly in the case of the molar ratio of (alkyne)/(azide) = 0.5/0.5. This may be due to the decomposition of nitro groups, which caused the decomposition of the polymeric matrix [17].

**Figure 7:**
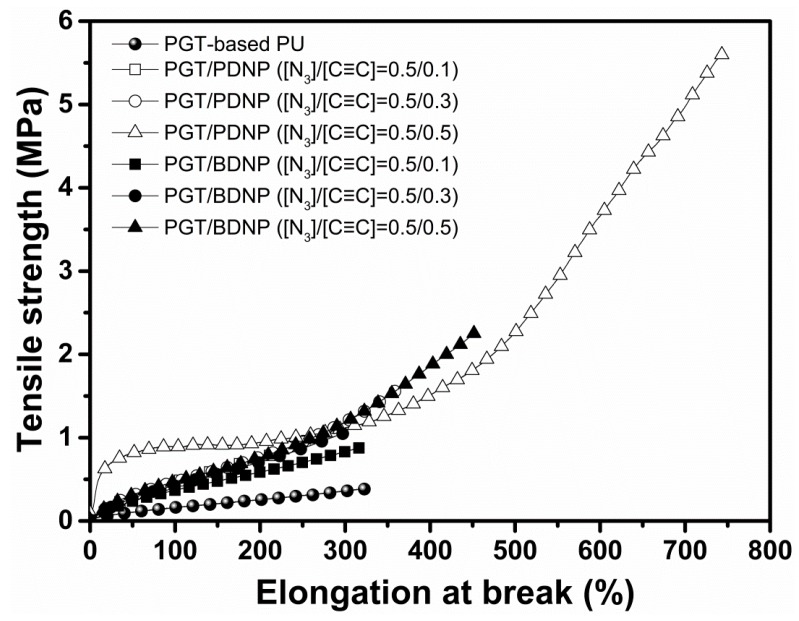
Mechanical properties of the control PGT-based PU and REP/PGT-based PUs.

Figure 7 presents the stress-strain curves of the control PGT-based PU and REP/PGT-based PUs. Compared to the control PGT-based PU, a low feeding of REP ((alkyne)/(azide) = 0.1/0.5) engendered an appreciable increase in tensile strength and modulus from 0.40 MPa to 0.88 MPa and from 0.21 MPa to 0.76 MPa, respectively. As the ratio of [alkyne]/[azide] was increased to 0.3/0.5, the tensile strength and modulus of the REP/PGT-based PUs were increased, from 0.99 MPa to 1.45 MPa and from 0.72 MPa to 0.82 MPa, respectively. These results showed that the REP/PGT-based PUs behaved as an elastomer. A dramatic increase in tensile strength from 2.22 MPa to 5.23 MPa and in modulus from 0.96 MPa to 6.71 MPa was observed at (alkyne)/(azide) = 0.5/0.5 where the specimen presented a stiffer and more ductile behavior. This indicated that a transition in the magnitude of intermolecular interaction within the REP/PGT-based PUs had changed significantly when the ratio of (alkyne)/(azide) was increased to 0.5/0.5. It also suggested that the elevated content of the resulting triazole groups via the 1,3-DPCA reaction in the pendant chain increased the steric hindrance and reduced the flexibility of the polymer chains, generally resulting in increased stiffness and polymer rigidity [26]. At (alkyne)/(azide) = 0.5/0.5, the PDNP-incorporated PUs exhibited a distinct stress-strain curve with a yielding process. The PDNP/PGT-based PU deformed elastically under the yield point (ca. 0.60 MPa) and started plastic deformation over the yield point. Beyond the yield limit, the stress increased with increasing elongation, suggesting that the specimen was subjected to strain-hardening behavior, because considerable elongation facilitated the orientation of the polymer chain [26]. In terms of the BDNP/PGT-based PU at (alkyne)/(azide) = 0.5/0.5, the specimen deformed elastically with a low yield point, and strain hardening was also observed during plastic deformation. Basically, these results would be an alternative to customizing the required mechanical properties of the energetic binders by adjusting the equivalent ratio of (alkyne)/(azide) for all binder systems.

Table 3 lists the thermal properties, density, calculated thermodynamic properties, sensitivities and detonation parameters of compounds **1**–**4**. The impact sensitivities were explored by BAM methods. The density of REP was obtained using an Anton Paar DMA 5000 density meter, and that of all compounds was calculated by the group contribution method [35]. The thermodynamic properties of the compounds were predicted through quantum chemical calculations. The structural optimizations of all compounds were calculated at the B3LYP/6-31+G(d,p) level of theory using the Gaussian 09 revision A.02 program package. To compare the energetic performance, detonation parameters were performed using the program package EXPLO5 (version 6.02) [36] based on the calculated liquid state heats of formation, as well as the measured and calculated densities.

Compared to the REPs, REP triazoles had a higher enthalpy of formation (Δ_f_ $H_{m}^{0}$ = −161.8 kJ mol^−1^ (**1**), −202.2 kJ mol^−1^ (**2**), 18.1 kJ mol^−1^ (**3**), −16.2 kJ mol^−1^ (**4**)). REP triazoles showed a higher energy of explosion (Δ_E_ *U*^0^ = −5034 kJ kg^−1^ (**1**), −4856 kJ kg^−1^ (**2**), −5046 kJ kg^−1^ (**3**), −4888 kJ kg^−1^ (**4**)) and explosion temperature (*T*_E_ = 3142 K (**1**), 2956 K (**2**), 3200 K (**3**), 3034 K (**4**)). REP triazoles displayed a higher detonation pressure (*p*_CJ_ = 13.9 GPa (**1**), 12.4 GPa (**2**), 17.0 GPa (**3**), 15.4 GPa (**4**)) and detonation velocity (*V*_Det_= 6430 m s^−1^ (**1**), 6189 m s^−1^ (**2**), 6979 m s^−1^ (**3**), 6751 m s^−1^ (**4**)). Moreover, REPs triazoles had a higher specific impulse (*I*_s_ = 208 s (**1**), 204 s (**2**), 213 s (**3**), 208 s (**4**)). Overall, the REP triazole species exhibited a superior performance to the pure REPs.

## 4. Conclusions

The *gem*-dinitro REPs with the terminal alkyne group were synthesized via Michael addition, hydrolysis reaction and esterification reaction, respectively. Hydroxy-telechelic PGT prepolymer was synthesized sequentially by cationic ring-opening polymerization and azidation reaction. The quantitative miscibility with respect to the *I* value of a binary mixture of PGT and REPs showed that the REPs with a lower *I* value were more miscible with the PGT prepolymer than EPs. The REPs could effectively reduce the viscosity of the PGT prepolymer from 1485 to 350~450 cP at 30 °C with a REP loading of up to 50 wt %, which was much lower than that of the PGT/EP mixtures. Both plasticization and viscosity reduction implied that the REPs could work efficiently as common plasticizers during processing. The 1,3-DPCA reactivity of propargyl versus 3-butynyl moiety mainly depended on the distance between the EWG and the neighboring alkyne, where an inductive effect alleviated the 1,3-DPCA reactivity. The *E*_a_ was determined to be 83.4 kJ/mol for PDNP/PGT, which was lower than the 91.6 kJ/mol observed for BDNP/PGT from DSC analysis. ^1^H NMR spectroscopy revealed the deficient electron density of the terminal alkyne to have originated from the degree of deshielding with the corresponding proton in the terminal alkyne of pure REPs. Both REPs are able to react completely with the polymer in 7 days, suggesting the benign compatibility within the PU polymerization and 1,3-DPCA reaction. PGT-based energetic PU binders with the incorporation of REP were prepared and characterized by FT-IR spectroscopy. TGA of all PUs showed a similar *T*_d,max_ of around 233 ± 4 °C, indicating that REP/PGT-based PUs maintained the thermal stability of the control PGT-based PU, and the REPs presented good thermal stability until the polymeric matrix decomposed. The REP/PGT-based PUs exhibited mechanical reinforcement, probably due to the increased steric hindrance and declined torsion of the polymer backbone originating from the resulting triazole groups via the 1,3-DPCA reaction in the pendant chain. In addition, the REP/PGT-based PUs presented higher tensile strength than REP/GAP-based PUs at the same REP content, suggesting that the tetramethylene oxide soft segment in the REP/PGT-based PUs played a dominant role in enhancing the mechanical properties. The impact sensitivity of the REP/PGT-based PUs was also weakened by the transforming sensitive azide group to the triazole group and was also lower than the REP/GAP-based PUs for the same REP content. Compared to the pure REPs, REP triazoles displayed higher energetic performance in terms of energy of explosion, explosion temperature, detonation pressure, detonation velocity and specific impulse.

## Figures and Tables

**Table 1:** Summary of the plasticization effect of REPs and EPs on the PGT prepolymer.

	*I* Value (K)	Viscosity (cP) ^a^	
		30 °C	60 °C
PGT: REPs			
PGT:PDNP PGT:BDNP	6.31 ± 0.34 3.01 ± 0.33	450 350	140 114
PGT: Conventional EPs			
PGT:BDNPF/A ^b^ PGT:BDNPF/BF ^c^	11.04 ± 0.56 10.52 ± 0.50	692 648	160 142

^a^ mixture of PGT:plasticizer (50:50 *w*/*w*) measured, PGT prepolymer (1485 cP at 30 °C and 306 cP at 60 °C); ^b^ BDNPF/BDNPA, ^c^ BDNPF/BDNBF.

**Table 2:** Summary of thermal stabilities and mechanical properties of REP/PGT-based PUs.

PU Binders	Mole Ratio of [C≡C]/[N_3_]	TGA in N_2_		Mechanical Properties		
		*T*_d,5wt%_ (°C)	*T*_d,max_ (°C)	Tensile Strength (MPa)	Elongation at Break (%)	Modulus (MPa)
PGT PU	0/0.5	227	237.1	0.40 ± 0.04	325 ± 40	0.21 ± 0.01
PDNP (*n* = 1) PGT-based PU	0.1/0.5	221.7	234	0.87 ± 0.06	246 ± 17	0.76 ± 0.01
	0.3/0.5	222	233.3	1.45 ± 0.18	347 ± 23	0.82 ± 0.02
	0.5/0.5	227	229.7	5.23 ± 0.33	713 ± 34	6.71 ± 0.73
BDNP (*n* = 2) PGT-based PU	0.1/0.5	220.3	236	0.88 ± 0.06	324 ± 24	0.59 ± 0.02
	0.3/0.5	221	229.7	0.99 ± 0.05	280 ± 16	0.72 ± 0.01
	0.5/0.5	230	229.7	2.22 ± 0.03	432 ± 19	0.96 ± 0.07

**Table 3:** Basic properties and detonation parameters of **1**-**4**.

	1	2	3	4
Structure	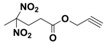	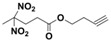	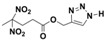	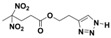
Formula	C_8_H_10_N_2_O_6_	C_9_H_12_N_2_O_6_	C_8_H_11_N_5_O_6_	C_9_H_13_N_5_O_6_
FW (g mol^−1^)	230.18	244.20	273.20	287.23
*N*^a^ (%)	12.17	11.47	25.63	24.38
*O*^b^ (%)	41.70	39.31	35.14	33.42
*T*_d,onset_^c^ (°C)	191.0	199.5	−	−
*T_g_* (°C)	−78.71	−80.21	−	−
(g cm^−3^)	1.2945 ^d^	1.2691 ^d^	1.4892 ^e^	1.4497 ^e^
_f_$H_{m}^{0}$^f^ (kJ mol^−1^)	−161.8	−202.2	18.1	−16.2
_f_$U_{m}^{0}$^g^ (kJ g^−1^)	−0.70	−0.83	0.07	−0.06
IS ^h^ (J)	54.6	54.6	−	−
Explo5 V6.02 values				
_E_*U*^0 i^ (kJ kg^−1^)	−4930	−4764	−5046	−4888
*T*_E_^j^ (K)	3143	2952	3200	3034
*p*_CJ_^k^ (GPa)	11.7	10.6	17.0	15.4
*V*_Det_^l^ (m s^−1^)	6020	5815	6979	6751
Gas vol. ^m^ (L kg^−1^)	580	594	514	529
*I*_s_^n^ (s)	208	204	213	208

^a^ nitrogen content. ^b^ oxygen content. ^c^ temperature of decomposition by DSC (onset values). ^d^ density by measurement. ^e^ density by group contribution method. ^f^ molar enthalpy of formation. ^g^ energy of formation. ^h^ impact sensitivity. ^i^ energy of explosion. ^j^ explosion temperature. ^k^ detonation pressure. ^l^ detonation velocity. ^m^ gas volume assuming only gaseous products. ^n^ specific impulse.

## Supplementary Materials

The supplementary materials are available online at http://www.mdpi.com/2073-4360/10/5/516/s1.
