# Supplementary material for: Reactive Energetic Plasticizers Utilizing Cu-Free Azide-Alkyne 1,3-Dipolar Cycloaddition for In-Situ Preparation of Poly(THF-co-GAP)-Based Polyurethane Energetic Binders
Source: Polymers (Basel). 2018 May 10;10(5):516. doi: 10.3390/polym10050516 (PMC6415412; doi:10.3390/polym10050516)
Supplement: Supplementary file 1 [file polymers-10-00516-s001.pdf]

Supplementary Materials

Reactive energetic plasticizers utilizing Cu-free azide-alkyne 1,3-dipolar cycloaddition for in-situ preparation of poly(THF-co-GAP)-based polyurethane energetic binders

Mingyang Ma[1] and Younghwan Kwon[2,*]

[1]Jiangxi Province Key Laboratory of Polymer Micro/Nano Manufacturing Devices, East China University of Technology, Nanchang 330013, People's Republic of China; mmy861201@163.com (M.M.)

[2]Department of Chemical Engineering, Daegu University, Gyeongsan, Gyeongbuk 38453, Republic of Korea

* Correspondence: y_kwon@daegu.ac.kr; Tel: +82-53-850-6569

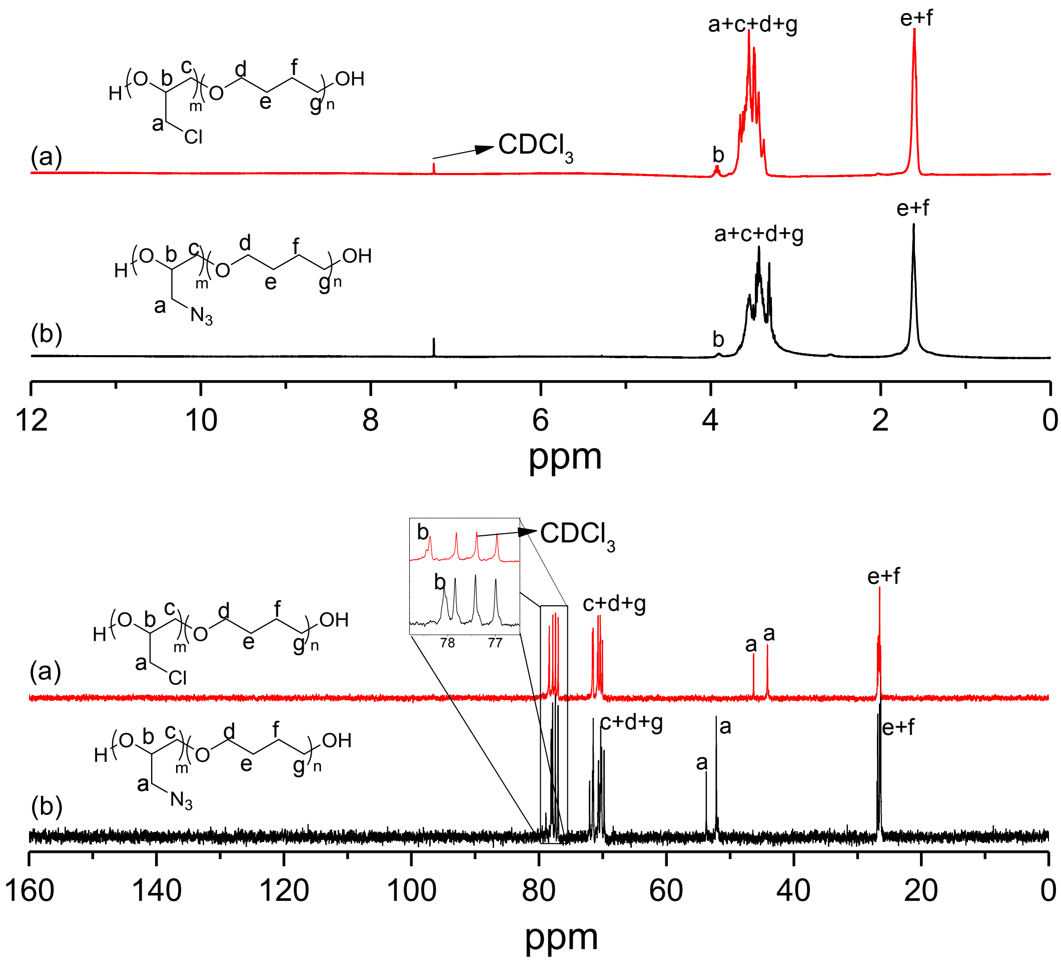

Fig. S1. ^{1}H (top) and ^{13}C NMR (bottom) spectra of (a) poly(ECH-co-THF) and (b) poly(GAP-co-THF).

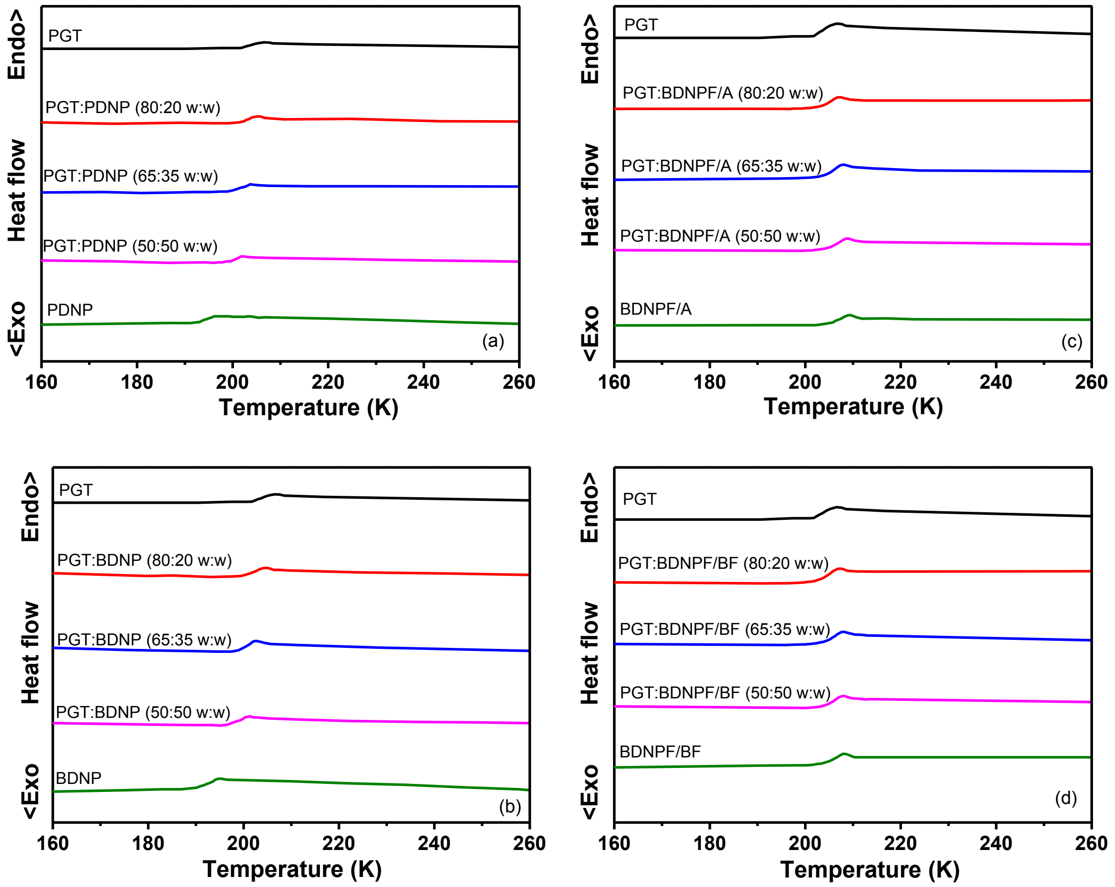

Fig. S2. DSC thermograms of (a) PGT:PDNP, (b) PGT:BDNP, (c) PGT:BDNPF/A, and (d) PGT:BDNPF/BF.

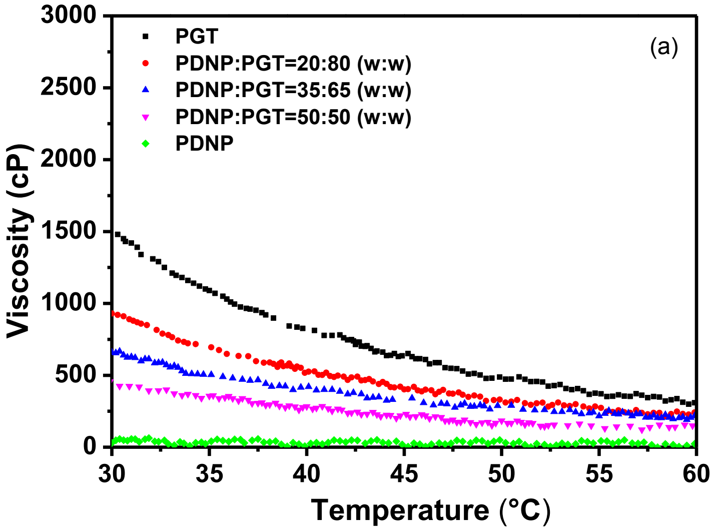

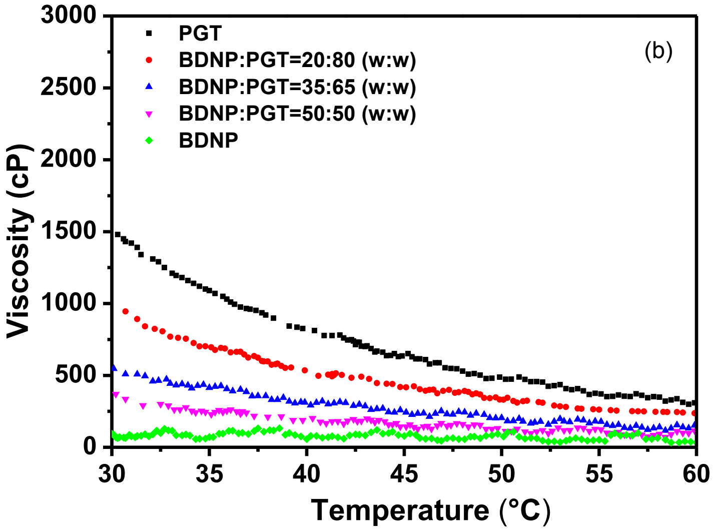

Fig. S3. Viscosity reduction of (a) PDNP : PGT, and (b) BDNP : PGT

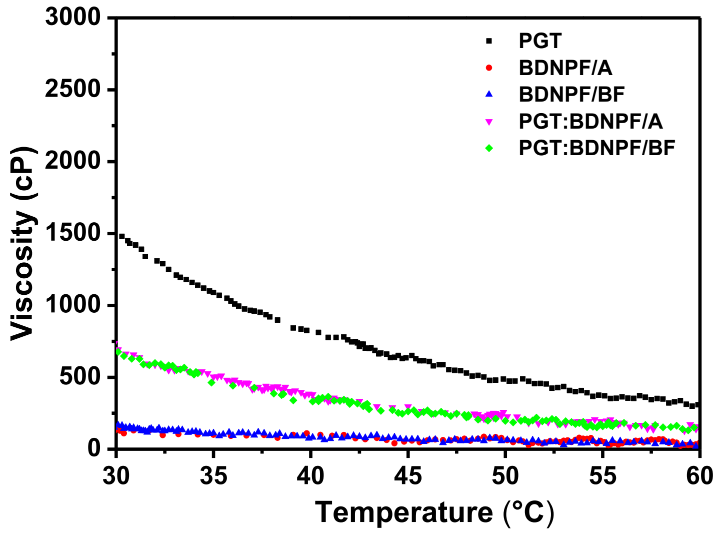

Fig. S4. Viscosity reduction of PGT prepolymer plasticized with 50 wt% of conventional EPs.

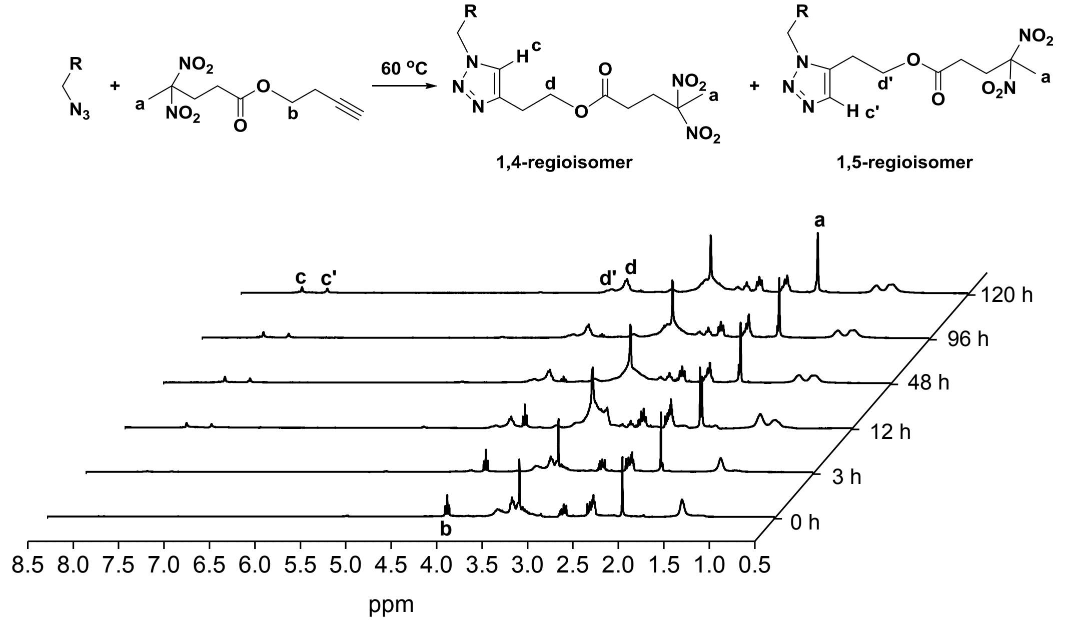

Fig. S5. ^{1}H NMR spectra as a function of reaction time of Cu-free azide-alkyne 1,3-dipolar cycloaddition reaction of BDNP (n=2) and PGT prepolymer carried out in bulk condition at 60 °C. The R group in the reaction scheme above is the backbone of PGT prepolymer.

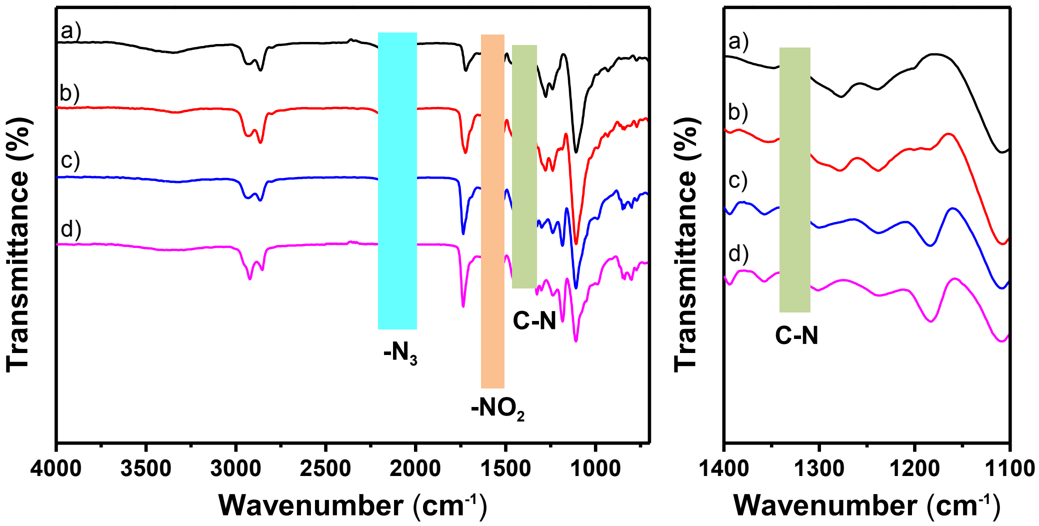

Fig. S6. FT-IR spectra of the BDNP/PGT-based PUs in terms of [C≡C]/[N_3] (mol/mol): a) 0/0.5, b) 0.1/0.5, c) 0.3/0.5 and d) 0.5/0.5.

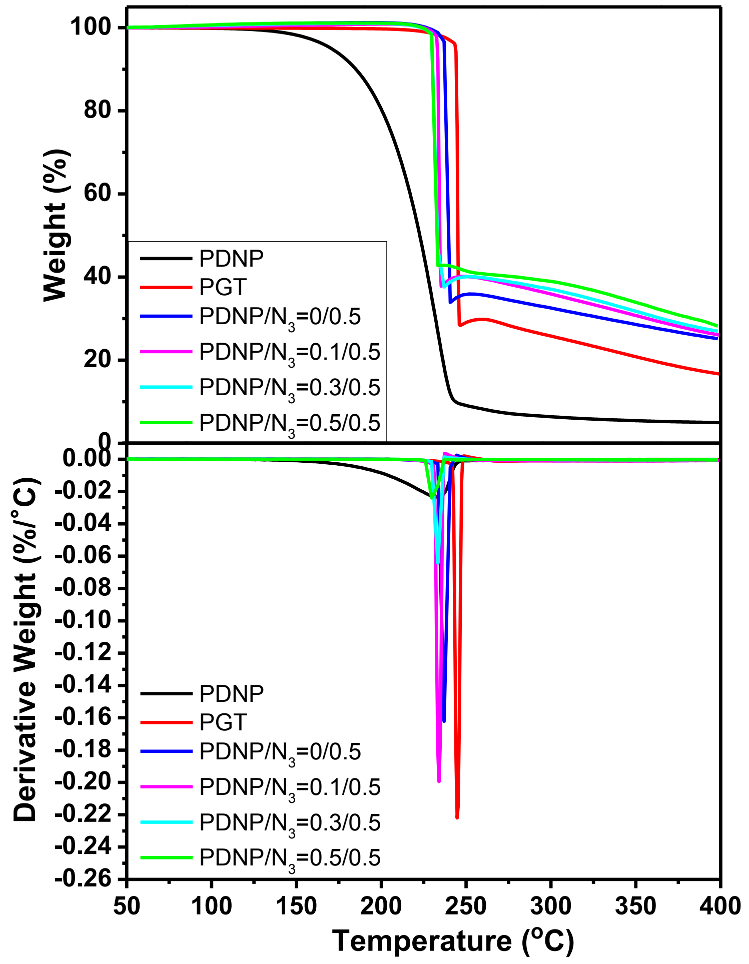

Fig. S7. TGA (top) and DTG (bottom) curves of the PDNP/PGT-based PUs.

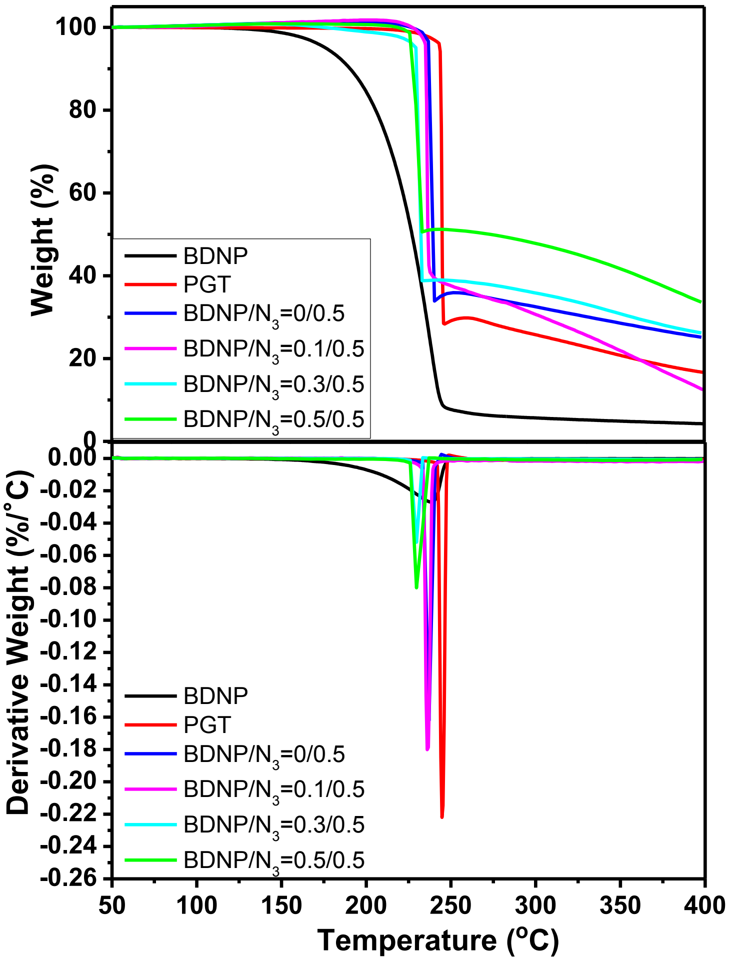

Fig. S8. TGA (top) and DTG (bottom) curves of the BDNP/PGT-based PUs.
